# Modelling human papillomavirus biology in oropharyngeal keratinocytes

**DOI:** 10.1098/rstb.2018.0289

**Published:** 2019-04-08

**Authors:** Sally Roberts, Dhananjay Evans, Hisham Mehanna, Joanna L. Parish

**Affiliations:** Institute of Cancer and Genomic Sciences, College of Medical and Dental Sciences, University of Birmingham, Vincent Drive, Birmingham B15 2TT, UK

**Keywords:** human papillomavirus, head and neck cancer, human papillomavirus life cycle, tonsil keratinocytes, oropharyngeal cancer

## Abstract

Most human papillomavirus (HPV) positive head and neck cancers arise in the tonsil crypts; deep invaginations at the tonsil surface that are lined with reticulated epithelium infiltrated by immune cells. As in cervical HPV infections, HPV16 is the most prevalent high-risk type in the oropharyngeal cancers, and a genital-oral route of infection is most likely. However, the natural history of HPV-driven oropharyngeal pathogenesis is an enigma, although there is evidence that it is different to that of cervical disease. It is not known if the virus establishes a productive or abortive infection in keratinocytes of the tonsil crypt, or if viral infections progress to cancer *via* a neoplastic phase, as in cervical HPV infection. The HPV DNA is more frequently found unintegrated in the cancers of the oropharynx compared to those that arise in the cervix, and may include novel HPV-human DNA hybrids episomes. Here, we review current understanding of HPV biology in the oropharynx and discuss the cell-based systems being used to model the HPV life cycle in tonsil keratinocytes and how they can be used to inform on HPV-driven neoplastic progression in the oropharynx.

This article is part of the theme issue ‘Silent cancer agents: multi-disciplinary modelling of human DNA oncoviruses’.

## 1. Human papillomavirus-driven cancer in the oropharynx

In the United Kingdom (UK), the age-standardized incidence rate of oropharyngeal squamous cell carcinoma (OPSCC) has tripled in men and doubled in women between 1995–2011 and similar trends have been reported in the United States (US), and in other parts of the world [1,2]. A human papillomavirus (HPV) infection is a significant contributing factor to the rising trend and is notably associated with cancers of lymphoepithelial sites within the oropharynx; specifically within the tonsil and the base of tongue [1,3–6]. Intriguingly, some studies have shown a levelling out of HPV prevalence in tumours post-2002, suggesting factors other than HPV may be driving the increase in OPSCC in later years [1,7]. The worldwide burden of OPSCC attributable to HPV is estimated to be a third of all OPSCC cases (29 000 cases per year). There is a greater incidence of HPV-positive OPSCC in the developed rather than less developed countries, with North America and Europe with the highest number of cases [8]. These virus-associated cancers were found to be more common in young (less than 60 years), male patients, although more recent studies identified expansion of the elderly population (greater than 70 years) with HPV-positive disease [9]. Sexual behaviour measured as lifetime number of oral sexual partners is a very strong risk factor for HPV-OPSCC, indicating that an genital-oral route for oral HPV infection is most likely [2,10,11]. Smoking may also have a biological effect upon the natural history of oral HPV infection and OPSCC development since this behaviour has been linked to an increase in acquisition and/or persistence of oral HPV infection [12–14].

There is much interest in patients with HPV-OPSCC because they have overall increased survival compared to those with HPV-negative disease, and thus it has been proposed that this group of younger patients could benefit from deintensification of therapy to reduce the longer term toxicities in anticipation of long-term survival [15,16]. However, two recent clinical trials have demonstrated that treatment deintensification in low-risk HPV-OPSCC from cisplatin-based chemoradiotherapy to cetuximab bioradiotherapy did not result in reduced toxicity and the deintensified patients had reduced overall survival [17,18]. Also, despite the better survival of patients with HPV-positive tumours there is a significant minority of patients that respond poorly to treatment and have a poor prognosis. While reduced treatment response in this group of patients has been correlated with heavy cigarette smoke exposure [15,19], the oropharynx sub-site origin of the tumour determines clinical outcome [3]. Thus, tumours that have arisen from the lymphoepithelial sites of the tonsil and base of tongue show beneficial survival compared to HPV-negative tumours at these sites, but patients with HPV-positive tumours originating from oropharyngeal sites outside of the lymphoepithelial tissues have no advantage in outcome compared to virus-negative cancers [3]. Nevertheless, there is currently no widely accepted strategy to identify patients that will respond better to treatment. However, HPV-OPSCC tumours with low numbers of tumour-infiltrating lymphocytes (TIL) have a poorer prognosis, and this could be used to predict those patients that will respond poorly to treatment [20].

The high-risk (HR) HPV types found in cervical cancers are also present in OPSCC and HPV16 is the most prevalent HR type in both types of tumour [21]. However, a far greater proportion (over 80%) of OPSCC are HPV16-positive compared to cervical tumours in which approximately 60% are HPV16-positive [22]. In addition, the frequency of HPV18 is far less in OPSCC (less than 2%) than in cervical disease (10%); and in fact, HPV33 is the second most prevalent HR type in OPSCC (3.3%) [21,23]. The reasons for HPV16 dominance over the other HR viruses in OPSCC is not clear, but could reflect the nature of the target cell of infection within the tonsil, which may be different between HPV16 and the other HPV types, differences between the infectious cycles or the interaction of these viruses with the host immune response. With regard to the latter possibility, primary foreskin keratinocytes immortalized by HPV16 are sensitive to tumour necrosis factor alpha treatment, whereas HPV18-immortalized cells are resistant to this inhibitory cytokine [24]. This is in part owing to different cellular gene expression changes induced by HPV16 and HPV18, which may contribute to the differences in the natural history of cervical pathogenesis between these two viruses [25,26]. However, little is known about how HPV infection in the tonsil manipulates the immune response, or whether there are differences between HPV types at this anatomical site.

Chromosomal integration of HPV genomes is a common feature of cervical cancers and at this body site is linked to a poorer clinical outcome. Integration of the viral DNA most often disrupts the E1 and E2 genes and deregulates virus gene transcription, leading to maintained and often high expression of the HPV oncoproteins E6 and E7 in the tumours. However, in HPV-OPSCC the primary form of HPV DNA is non-integrated episomes and viral DNA integrants are less frequent than some earlier studies indicated; most likely owing to a combination of the use of different methodologies to detect episomes and/or integrants, and ambiguities in data interpretation [27–29]. As in cervical cancer, HPV DNA integration and a loss of expression of the viral regulator E2 is linked to worse clinical outcomes in OPSCC [7,30]. More recent genome-wide analyses have led to some intriguing findings in relation to the structure of HPV DNA in HPV-positive tumours. Work from the Gillison laboratory [31] indicated that HPV16 DNA integration was linked to insertional mutagenesis in head and neck cancer, and cervical cancer cell lines. The viral integrants were flanked by a myriad of genomic structural variations and such amplifications and rearrangements were present in HPV-positive head and neck tumours. They proposed that these structural alterations occurred through viral origin-directed replication of HPV16 integrants and through a process of recombination and looping formed structural rearrangements and viral-host sequence concatemers. A third form of HPV DNA has been proposed by Morgan and colleagues to be present in OPSCC—a circular, extrachromosomal HPV-human DNA hybrid in which the HPV genome is a multimer (dimer or greater), but some of the copies carry viral DNA deletions and human DNA sequences [32]. The structure of these HPV-human DNA hybrids suggests that during the natural history of the infection, the viral DNA was integrated into the host DNA and through an unknown mechanism was subsequently excised and able to replicate, most likely in an E1-E2 dependent manner, as an HPV-human chimeric episome [29]. A human-HPV16 chimera has been reportedly found in a cervical cancer so perhaps these structures might not be unique to the head and neck [33]. It is interesting that these host–virus chimeric episomes were proposed in the Gillison study [30] as a transient intermediate, replicated by rolling circle amplification, that eventually form integrated concatemers with identical virus–host and virus–virus breakpoints.

Recent analysis of the mutational landscape of HPV-positive (predominantly OPSCC) in comparison to HPV-negative (predominantly oral cavity) squamous cell carcinomas provides evidence that the mutation burden in HPV-positive tumours is indicative of apoliprotein B mRNA editing enzyme, catalytic polypeptide-like (APOBEC) cytidine deaminase editing [34]. Interestingly, the mutational burden with HPV-positive tumours was similar regardless of smoking status, suggesting that the APOBEC-mediated innate anti-viral responses are the main driver of genetic mutations in these tumours, although it has been clearly demonstrated that tobacco use reduces treatment success [15], which could reflect an attenuated immune infiltrate induced by tobacco exposure. Furthermore, the mutational burden in HPV-positive tumours was significantly distinct from HPV-negative tumours which displayed a more broad mutational signature distribution that is reflective of heavy tobacco and alcohol use. The APOBEC-specific mutational signature of HPV-OPSCC has also been identified in HPV-driven cervical cancers [35] and several of the frequently mutated genes appear to be consistently identified at both body sites including *PIK3CA*, *EP300*, *PTEN*, *FBXW7* and *TGFBR2*. However, several other genes found to be commonly mutated in HPV-OPSCC, including *ZNF750*, *FGFR3*, *CASZ1*, *CYLD* and *RIPK4* have not been identified in cervical cancer [34], indicating that the biological behaviour of HPV and natural history of HPV-driven carcinogenesis is different at distinct anatomical sites.

### (a) Does the target cell for human papillomavirus infection reside in the tonsil crypt?

**Figure 1.**
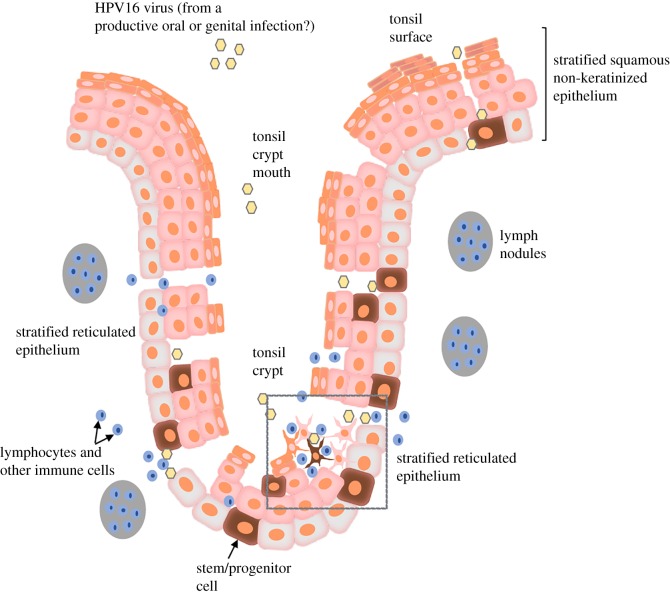
The tonsil epithelium. A schematic of the tonsil highlighting the difference in the epithelia lining the tonsil crypt surface (stratified squamous non-keratinized) and the crypt (stratified reticulated). The boxed region depicts the keratinocytes typical of the crypt reticulated epithelia, with long slender processes interconnected with neighbouring cells through desmosome junctions. The intercellular spaces are filled by infiltrating lymphocytes. See text for details.

The mucosa of the tongue base and tonsils (palatine and lingual) is invaginated and forms crypts lined with reticulated epithelium (figure 1). It is within these crypts that HPV-positive tumours consistently arise, in contrast to HPV-negative lesions that emerge mainly from the epithelium lining the tonsil surface [3,36]. Unlike the stratified, non-keratinizing epithelium of the surface of the tonsil, which is polarized and undergoes morphological maturation, the epithelium of the crypt is devoid of a continuous basal lamina between the epithelium and the underlying lymphoid stroma, and undergoes incomplete differentiation [37,38]. The crypt keratinocytes have long slender processes that form a desmosome-mediated network with intercellular spaces filled with the lymphoid cells (figure 1). The crypts are also characterized by an abundant network of intraepithelial blood vessels [39]. The specificity of HPV tumours for such an immunologically alert anatomical site is intriguing [40]. The disrupted nature of this lymphoepithelium may enable the virus to readily gain access to the exposed basal keratinocytes and as an immune privileged site (high levels of the lymphocyte inhibitor PD-L1 are found in the tonsil crypts, but not in the tonsil surface epithelium [41]), the virus may evade immune detection and clearance to establish a persistent infection. However, immune presentation must occur as HPV16 E6 seroconversion arises at least 10 years before diagnosis of OPSCC [42]. Also, the favourable clinical outcome of these virus-positive tumours has been linked to a strong host immune response with high frequency of infiltrating TIL and inflammatory responses observed, although in some patients a trigger of the PD-1:PD-L1 checkpoint may act to limit the capacity of the TIL to eliminate the tumour [20,41].

Analysis of non-malignant tonsil tissue for indications of HPV infection suggest that HPV infection at this site is rare, even when the reticulated epithelia was sampled [43]. This rather puzzling finding could also mean that HPV infection is of a focal nature and limited to a small number of cells in the crypt and hence difficult to detect. Since the HPV life cycle is dependent on epithelial maturation for completion; the nature of the reticulated squamous epithelia of the crypt may not be permissive for HPV replication. Thus, if HPV infection at this site most commonly leads to an abortive infection with deregulation of early viral gene expression, these cells might be at high risk of immortalization and malignant transformation.

Mucosal squamous epithelia at different anatomical sites that are susceptible to HPV infection are at different risks of cancer development. The uterine cervix has a nearly 100% association with HR HPV whereas at other sites including anus, vagina/vulva and oropharynx, only a subset of the tumours are HPV-associated. This differential risk between mucosa sites could be explained by the nature of the target cell for HPV infection, which influences disease outcome. In the cervix, a small and discrete population of non-stratified cuboidal cells of embryonic origin in the metaplastic transformation zone at the squamocolumnar junction (SCJ) are highly susceptible to HPV infection and from which the majority of neoplasms arise [44]. The SCJ cells have a specific embryonic expression profile and this expression profile is also expressed in cervical neoplasms [45]. These cells may provide an environment that is inhibitory/abortive to the normal replication cycle of the virus leading to deregulated HPV oncoprotein expression, as well as being a site of altered adaptive immunity to enable viral persistence [46]. While a similarly unique population of cells are found at the SCJ between the oesophagus and gastric epithelia (and could be cell origin of Barrett's oesophagus), a similar cellular makeup of the tonsil has not been identified. However, one of the SCJ biomarkers—cytokeratin 7 (CK7)—is expressed in the tonsil crypt, but less so in the surface squamous epithelia. Notably, CK7 expression was significantly associated with cancers of the tonsil site and that were HPV positive [47]. Thus CK7 positive tonsil cells may represent cells vulnerable to HPV16 infection and transformation. Intriguingly, CK7 and its binding partner cytokeratin 19 (CK19) have been shown to be potential regulators of HPV16 E7 protein levels; CK7 binds E7 transcripts to protect them from degradation and CK19 alleviates a translational block imposed by CK7 [48,49]. It is worthy to note that in the anal SCJ while multi-layers of CK7-positive cells make up the metaplastic transition zone, only a small proportion of HPV-positive anal neoplasms express CK7, suggesting that CK7 positive anal cells are less susceptible to HPV infection and/or have a reduced risk of neoplastic conversion [50].

Cervical cancer pathogenesis is characterized by a well-defined premalignant phase of low-grade to high-grade intraepithelial neoplasias (CIN1 to 3) that develop over many years, but dysplastic lesions have rarely been reported in the oropharynx. This, combined with lack of detection of HPV productive infections at this anatomical site, the dominance of HPV16-positivity and the difference in integration rates between the two sites suggests that the natural history of HPV-driven disease in the oropharynx is very different to the cervix, but as yet unexplained [51].

## 2. Modelling human papillomavirus pathogenesis in the tonsil

One approach that has proved very successful in understanding the interaction between HR HPV types and cervical keratinocytes has been by the analysis of cells isolated from low-grade cervical neoplasias. The most highly researched are HPV16-positive W12 cells [52] and the HPV31-positive CIN612 cell line [53]. Both harbour the viral genome as extrachromosomal episomes and upon organotypic raft culture form reconstituted epithelia resembling the low-grade cervical lesion from which they were derived. Notably, the cell lines show neoplastic progression upon extended culture; the viral DNA integrates and in organotypic growth they form a higher grade dysplastic epithelium [54–56]. While these cell lines have been invaluable in understanding cervical pathogenesis, as yet no such premalignant cell lines have been isolated from the oropharynx. Therefore, in this section, we discuss how HPV infection and disease progression can be modelled in the tonsil.

### (a) Transgenic mice

Transgenic mouse models engineered to express the HPV16 oncoproteins E6 and E7 in squamous epithelia and treated with chemical carcinogens have been very informative of the role of the viral oncoproteins to the development of skin, cervical, and head and neck (tongue and oesophagus) carcinogenesis [57–60]. These studies have shown that the nature of the contribution of the E6 and E7 proteins to carcinogenesis is not equal between the different anatomical sites, highlighting the importance of understanding E6 and E7 biology at the correct physiological site. However, because mice lack a tonsil equivalent, a similar approach to dissect E6 and E7 function in this lymphoid organ is not possible.

### (b) Tonsil tissue explants: *ex vivo*

One approach that as yet has not been exploited for investigating HPV-tonsil interactions is the use of tonsil explants. Fresh human tonsils dissected into small blocks can be cultured on collagen rafts at the liquid–air interface [61]. Tonsil explants have been used to study infection by viruses including human immunodeficiency virus 1 and Epstein-Barr virus [62]. Although these explants have a limited lifespan and can have extensive inter- and intra-experimental variation, one advantage of the tonsil explants is that the *ex-vivo* model maintains tissue cytoarchitecture, including the major lymphocyte subtypes and follicular dendritic network, and would enable HPV infection to be interrogated in the presence of a physiological immunological landscape.

### (c) Tonsil epithelium equivalents

**Figure 2.**
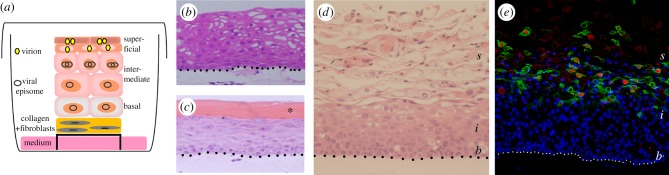
Epithelial equivalents. (*a*) Cartoon of growth of tonsil keratinocytes harbouring HR HPV episomes in organotypic raft culture. Stratification for two weeks at the liquid–air interface supports the complete productive HPV life cycle. (*b*) Haematoxylin and eosin stained section of primary tonsil keratinocytes and (*c*) foreskin keratinocytes. Note the formation of the keratinized corneum (*) in foreskin keratinocyte rafts that is absent from the rafts formed from primary tonsil keratinocytes. (*d*) Haematoxylin and eosin stained section of primary tonsil keratinocytes harbouring HPV16 episomes (b, basal, i, intermediate and s, superficial layers) and (*e*) immunostained for markers of the productive HPV life cycle: E4 protein (green) and the major capsid protein L1 (red). Nuclei are shown in blue. Scale bar, 20 µm. Dotted lines indicate the boundary between fibroblast-embedded collagen matrix and basal keratinocytes. Details of the methods involved in preparing the primary keratinocytes, transfection of HPV genomes, growth in organotypic raft culture and immunostaining of formalin-fixed, paraffin-embedded sections are given in [63].

Another approach and one that has been used extensively in the papillomavirus field is the generation of ‘HPV infected’ epithelium equivalents from primary keratinocytes grown in three-dimensional (3D) organotypic raft culture. Keratinocytes harbouring the viral genomes are seeded onto a dermal equivalent formed from rat tail type 1 collagen embedded with fibroblasts (mouse 3T3-J2 fibroblasts are routinely used in the HPV replication model). After a short period to allow expansion of the keratinocytes into a confluent monolayer on the collagen plugs, they are lifted onto a solid support and cultured at the air-liquid interface for a period of about two weeks—the time it takes for a basal keratinocyte to fully differentiate. This involves feeding the rafts from underneath with serum-containing medium that is free of epidermal growth factor, providing nutrients and allowing cytokines and growth factors produced from the fibroblasts to be delivered to the keratinocytes. The ‘raft’ system enables analysis of the spatial and temporal organization of the virus life cycle—from early events to viral progeny assembly (figure 2*a*).

Establishment of HPV genome-containing keratinocytes has largely relied upon transfection of HR HPV genomes along with a drug selectable marker into primary keratinocytes, followed by a brief period of drug selection to remove non-transfected cells [64]. The lifespan of the primary keratinocytes is extended by the actions of the E6 and E7 proteins, leading to immortalization of the cells and thus establishment of cell lines harbouring the viral episomes—a process that takes several months of cell culture. However, dependence on cellular immortalization prohibits investigation of immediate early events of initial viral genome amplification and the subsequent transition to maintenance replication, analysis of E6 and E7 immortalization defective genomes and characterisation of virus-cell interactions that precede immortalization. Modification of this model by using a Cre-loxP recombination system to recombine HPV episomes in primary cells—the loxP sites are positioned in the upstream regulatory region downstream of the late polyA+ site but in a region devoid of known binding factors—enables analysis of early events in the HPV life cycle [65–67]. More recently, primary keratinocytes were successfully infected with HPV16 pseudovirions which had first been conditioned on an extracellular matrix laid down by keratinocytes expressing the transient receptor laminin 332, and the infected primary keratinocytes supported the complete life cycle upon 3D culture [68].

Several groups including our own, are using primary tonsil keratinocytes to investigate HPV biology at this site (figure 2). Because of the very high prevalence of HPV16 in tonsil malignancies, the tonsil models have understandably focussed on HPV16. However, there is some sense in establishing tonsil models of the HR HPV types (e.g. HPV18) that are far less prevalent, because comparative analyses in isogenic cells may uncover clues as to reasons for the dominance of HPV16.

#### (i) Crypt versus surface squamous tonsil keratinocytes

One important question is why the tonsil crypt is more susceptible to HPV transformation than the surface squamous epithelium. A very interesting study isolated tonsil progenitor cells; cells expressing high levels of cell surface antigens CD44 and nerve growth factor receptor (NGFR) from the crypt and the surface epithelium [69]. In surface epithelium of the tonsil, CD44+/NGFR+ cells are in the basal and parabasal cell layers, but in the crypt they are found in a more random distribution, indicating the disrupted nature of the crypt (figure 1). The progenitor cells from both sites were molecularly similar (enriched for pathways associated with mobility and migration), but those from the crypt were more primitive than those of the surface based on their greater proliferative potential. While the lifespan of these cells from both sites was extended to similar extents upon co-expression of the HPV16 E6 and E7 oncoproteins, differentiation of the E6/E7 expressing crypt cells following 3D stratification was more markedly disrupted compared to the oncoprotein-expressing surface epithelial derived progenitors. Whether the progenitor cells are the natural target cell of the virus is not known but the more primitive nature of those from the crypt may render them less permissive to support the HPV16 life cycle and hence more susceptible to virus-driven transformation. Perhaps introducing whole HPV16 genomes into these cells to determine if they can support the infectious cycle would be one route to take.

#### (ii) Can HPV16 infect and replicate in tonsil keratinocytes?

HPV16 does replicate in ‘unsorted’ primary tonsil keratinocytes following transfection of complete viral genomes or infection with HPV16 pseudovirions [68,70,71]. Upon stratification, the HPV16 positive cells support the production of infectious progeny, although the conformation of the mature virions produced in the tonsil rafts differ to those assembled in keratinizing foreskin keratinocyte rafts [71]. The tonsil-derived virions were neutralized effectively with anti-L1 antibodies, but neutralization was less efficient with anti-L2 antibodies. If indeed there are structural differences between HPV virions produced at different body sites, this could impact on the efficacy of any future use of neutralizing L2-based prophylactic vaccines [72].

#### (iii) Do tonsil keratinocytes support replication of the less prevalent high-risk human papillomavirus types in tonsil malignancies?

**Figure 3.**
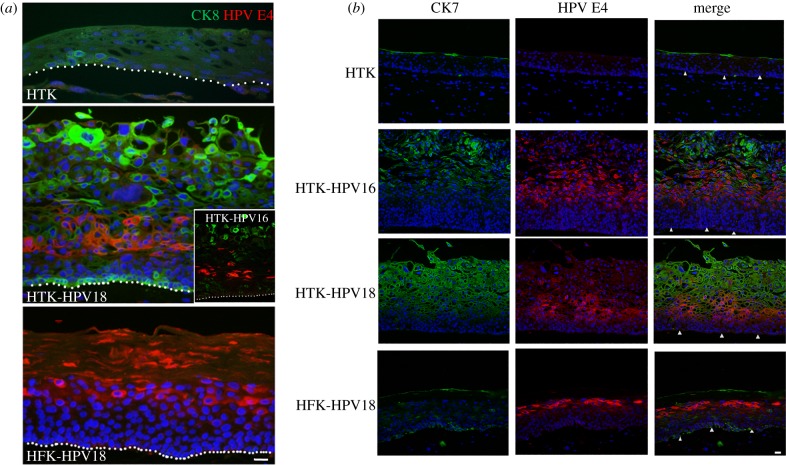
Cytokeratin expression in HPV-positive 3D rafts. Sections stained for (*a*) CK8 (Sigma-Aldrich, M20) and HPV E4, (*b*) CK7 (Dako Products, M701829-2) and HPV E4. Nuclei counterstained (blue). HTK, human tonsil keratinocytes; HFK, human foreskin keratinocytes. Scale bar, 20 µm. Dotted lines or arrowheads indicate the boundary between fibroblast-embedded collagen matrix and basal keratinocytes.

**Figure 4.**
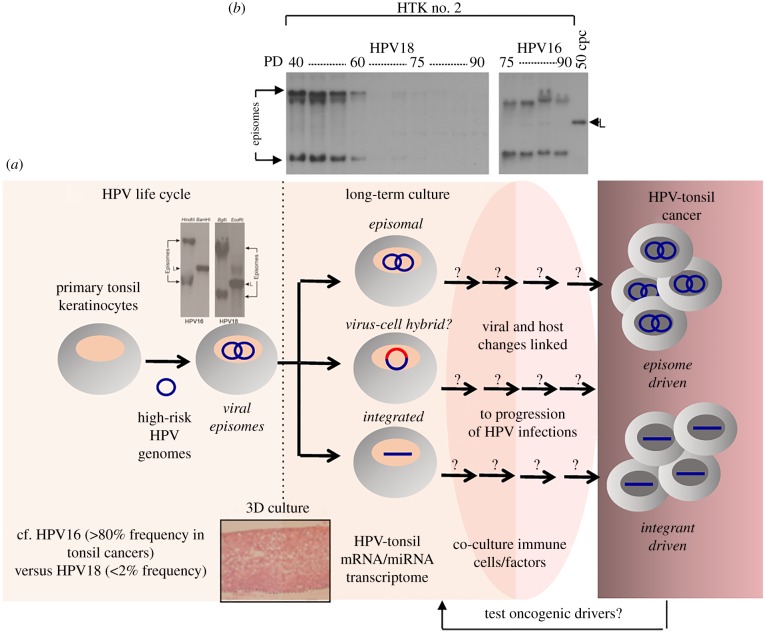
Tonsil equivalents to model HPV tonsil pathogenesis. (*a*) The primary tonsil keratinocytes harbouring HR HPV genomes are strong models to understand HPV biology at this anatomical site and the virus and host changes linked to early stages of disease progression. Also, they might be a platform to test the ‘driver’ capability of host gene mutations identified in HPV-driven tonsil cancers. Refer to text for details. (*b*) Southern blot of tonsil-HPV cells in extended two-dimensional cell culture shows episome loss and episome persistence; experimental details given in [63].

The rarity of other HR HPV types in oropharyngeal tissues might be because these types are unable to infect these tissues or they differ in replication potential. However, HPV18 is very effective in extending the lifespan of tonsil keratinocytes and the cells support HPV18 productive events upon 3D culture ([73] (figures 3 and 4)).

#### (iv) Are the primary tonsil keratinocyte-based models physiological?

When grown in organotypic raft culture, keratinocytes from squamous epithelia of different body sites exhibit an intrinsic potential for tissue-specific differentiation. Thus, keratinocytes isolated from neonate foreskin tissue form a cornified structure with a strong keratinized corneum, whereas tonsil keratinocytes recapitulate the structure of non-keratinizing epithelium (figure 2*b*,*c*). However, tonsil surface and crypt epithelia undergo alternative differentiation programmes and in particular, there are marked differences in their keratin profiles [74]. Cytokeratin 8 (CK8), normally partnered with CK18 is weakly expressed in keratinocytes of the surface tonsil epithelium (and in basal keratinocytes), but is strongly expressed in the reticulated crypt, particularly in the cells of the upper layers [74]. Another cytokeratin that distinguishes crypt from surface epithelia is the transitional/junctional epithelia marker CK7. In the surface epithelium, CK7 expression is low and patchy, whereas in the crypt it is strongly expressed [47].

So, do the tonsil epithelium equivalents show CK8 and CK7 expression patterns of the surface or the crypt, and does HPV replication affect the expression profiles of these cytokeratins? Our HR HPV tonsil keratinocytes models are beginning to answer these questions. Immunostained organotypic rafts of primary human tonsil keratinocytes using anti-CK8 antibodies showed patchy CK8 staining; it was largely absent from areas of full stratification, but in areas that were not full thickness, some basal/parabasal cells were positive for this cytokeratin (figure 3*a*). However, intense CK8 expression occurred in superficial cells of isogenic HPV16 and HPV18 tonsil epithelium equivalents, with low-level staining in basal keratinocytes (figure 3*a*). This was specific to the site of origin of the keratinocytes because very low CK8 staining occurred in rafts of foreskin keratinocytes harbouring the HR HPV episomes (figure 3*a*). In the tonsil keratinocyte rafts, CK7 was restricted to the occasional superficial cell, but in HPV16 and HPV18 genome-containing isogenic lines, the levels of this cytokeratin were markedly increased in cells of the upper intermediate and superficial layers corresponding to the productive phase of the HPV life cycle (figure 3*b*). A similar effect on CK7 expression did not occur in HPV18-positive foreskin keratinocyte equivalents (figure 3*b*). These findings provide evidence that HR HPV alters the expression pattern of tonsil crypt-specific differentiation markers, a phenomenon that appears to be unique to this anatomical site. HPV is known to delay differentiation in the cervix to support virus replication, but in the tonsil perhaps the virus is able to reprogramme the keratinocyte to express the differentiation characteristics of the tonsil crypt. Since CK7 has been implicated in a translational mechanism of HPV mRNAs [49], this might be a viral mechanism specific to the tonsil keratinocyte.

#### (v) Understanding neoplastic progression of human papillomavirus driven tonsil cancer using the human papillomavirus-tonsil models

Patients with HPV-positive oropharyngeal cancer often present with more advanced disease compared to those with HPV-negative OPSCC, but they have a better prognosis and overall survival, although recurrence is still a concern [15,75]. So while HPV positivity is a prognostic indicator, patient survival may be further stratified based on whether the viral genome is integrated or not in the tumour, since clinical outcome has been shown to be worse in those patients with integrated HPV16 and/or disruption of the HPV *E2* gene [7,16,30]. Identifying prognostic markers among these different populations of HPV-positive disease will be important if de-escalation treatment regimens are to be implemented successfully.

HPV DNA integration is far less frequent in HPV OPSCC than in the cervix; but in addition to integrated and unintegrated forms of the viral DNA, a third form of a viral DNA-human hybrid has been proposed [29]. Whether these viral-human hybrids truly exist and whether they are relevant for HPV-OPSCC pathogenesis is unclear, and as yet they have not been reported in tonsil keratinocytes immortalized by HPV16 genomes. However, HPV16 genomes do become integrated into host DNA in primary tonsil cells and the virus-cell E6-E7 mRNAs expressed are indistinguishable from those found in HPV16-positive head and neck tumours (figure 4), suggesting the same selective pressures occur in the tonsil equivalents as in clinical infections and thus, they represent good models in which to study virus-cell interactions in the progression of HPV-driven tonsil neoplasia [76].

Intriguingly, in our models of HPV replication in primary tonsil keratinocytes, in the three donors investigated to date, HPV18 is maintained as an episome for between 40 and 70 population doublings after which there is a marked decrease in episomal forms of the viral genome as determined by Southern blotting. Whereas in the same three donors, HPV16 DNA either integrated very early upon establishment of the cell lines (in one donor), and in the two other donors remained episomal in extended cell culture (greater than 90 population doublings to date). While the donor sample size is too small to conclude that HPV16 is less likely to integrate than HPV18 in primary tonsil keratinocytes, these models are useful to investigate differences in HPV16 and HPV18 biology and virus–host interactions in isogenic tonsil keratinocyte backgrounds grown in short and long-term cell culture under non-stress conditions (figure 4).

## 3. Future directions

It is a puzzle that despite the recognition of an association with HR HPV and OPSCC, infection at these sites in non-malignant tissue has not been conclusively found. Primary keratinocytes isolated from tonsil tissue are not refractory to HPV16 infection and are able to support the production of infectious progeny, but are the primary tonsil keratinocytes truly representative of the target cell for the virus? If stem cells in the crypt are the natural target and oral HPV infections may represent a source of virus to infect the tonsil, then these cells might be less permissive for HPV replication, leading to deregulated expression of E6 and E7. Since in the anogenital tract, the phenotype of the HR-HPV target cell is a determinant of carcinogenic risk, the molecular definition of the target cell of these viruses in the tonsil is needed [50].

The reason for the prevalence of HPV16 in HPV-OPSCC is also unclear; but primary tonsil keratinocytes support the infectious cycle of HPV18, that is rarely found in tonsil cancers, so simply that this HPV type is less able to infect or replicate in the tonsil cells seems an unlikely explanation of why we see less HPV18-positive tonsil cancers. Perhaps the nature of the interaction between these viruses and the immune system in this lymphoepithelial tissue is different between the various HPV types, and this contributes to the different outcomes of infection. Further studies to understand the effects of HPV infection and replication in tonsil keratinocytes upon innate signalling and other arms of the immune system are needed. With regards to this topic, RNA-Seq of gingivial keratinocytes immortalised with the telomerase enzyme hTERT and HPV16 genomes showed downregulation of the interferon-regulated innate signalling complex, interferon-stimulated gene factor 3 (ISGF3) and a number of the gene targets of this complex [70]. Although, gingivial tissue is not a common site for the development of HPV16 cancers, there was a strong correlation between the downregulation of the ISGF3 targets between these HPV16 immortalized cells and in HPV-positive versus HPV-negative head and neck cancers [70]. Levels of these targets were also low in tonsil keratinocytes harbouring HPV16 episomes, suggesting that interference of this pathway is key in HPV infections in the head and neck.

Investigation of the biology of the HPV life cycle in primary tonsils is one level of understanding of the virus–host interaction. However, since the tonsil is a lymphoidal tissue with significant infiltration of lymphoidal cells into the tonsil crypt, future studies might advance these models by co-culturing keratinocytes with immune components in two-dimensional and 3D culture systems.

While cell lines have been established from cervical neoplasias and have been of huge importance in understanding the natural history of cervical HPV pathogenesis, equivalents from the oropharynx have not been established. This might be because at this anatomical site the natural history of HPV infection is very different to the cervix as either a lengthy premalignant stage does not occur [51], or the dysplastic changes induced by replication of these viruses at this body site are very mild, as suggested for HPV18 replication in the cervix [77]. Nevertheless, during the culture of the HPV16-immortalized tonsil keratinocyte models, the viral DNA does integrate into host DNA and at chromosomal sites also found in HPV16-positive tumours, so they do model virus–host events that occur *in vivo* [76]. Our isogenic tonsil keratinocyte lines harbouring HPV16 or HPV18 show differential persistence of the viral episomes upon extended cell culture and, since the majority of HPV-driven cancers in the oropharynx retain episomes, they provide a useful model to investigate virus and cellular events associated with early stages of HPV integrant- and episome-driven tonsil pathogenesis (figure 4). Important outcomes might also include novel viral and/or cellular targets that could be developed into diagnostic and prognostic markers.

Performing global gene expression analysis on organotypic raft cultures of primary cervical keratinocytes harbouring HPV16 episomes revealed a number of host genes modulated by the virus (genes associated with cell cycle progression and DNA metabolism were upregulated whereas those genes involved in skin development, immune response and cell death were downregulated) that had not been observed in HPV16-positive foreskin based models [78]. The biology of the tonsil epithelium is very different to the cervix and global gene analysis on the 3D HPV16 tonsil epithelial equivalents are necessary to allow a better understanding the virus's reprogramming of the host at this body site. Because HPV regulates miRNA expression during the virus life cycle and a specific miRNA profile is associated with HPV-positive tonsil tumours compared to virus negative malignancies, then characterization of the virus’ effect upon the miRNA transcriptome should also be investigated using the models [79].

Neither should studies be limited only to HPV16, but include models harbouring HR HPV types found less frequently in HPV-OPSCC which might aid identification of the molecular reasons for the different pathogenicity in tonsil tissue.

It is commonly assumed that papillomaviruses evolve slowly, but next-generation sequencing of HPV DNA isolated from lesions is challenging this assumption and in fact inter-host HPV genotype variability might be high, although conservation of E7 has been demonstrated in the cervix [33,80,81]. There is also a degree of intra-host variability of HPV16 genotypes across anatomical sub-sites, suggesting that not all HPV16 infection sites are equal when it comes to viral diversity. But what factors could be driving HPV evolution at different infection sites? A large proportion of these mutations are driven by the anti-viral activity of APOBEC3. Other innate and adaptive immune pressures may also be important and differences between the cervix and oropharynx may, therefore, drive different levels of HPV genotype diversity. Certainly, this is an area of ongoing debate and more information about intra-host variability of the HPV16 genotype is required in HPV-OPSCC; the biology of any HPV16 variants unique to OPSCC could be tested in the primary tonsil keratinocyte models.

In summary, it is becoming recognized that virus-driven oropharyngeal disease is different to cervical pathogenesis: thus the refinement and enhancement of tonsil keratinocyte-based models will contribute to a greater understanding of HPV disease at this anatomical site.

## Data accessibility

The data supporting this article has been described in the text or are available upon request from the corresponding author.
